# Analysis and quantification of bone healing after open wedge high tibial osteotomy

**DOI:** 10.1007/s00508-019-01541-8

**Published:** 2019-09-09

**Authors:** Elena Nemecek, Catharina Chiari, Alexander Valentinitsch, Franz Kainberger, Gerhard Hobusch, Alexander Kolb, Lena Hirtler, Carmen Trost, Slobodan Vukicevic, Reinhard Windhager

**Affiliations:** 1 Department for Orthopedics and Trauma-Surgery, Medical University of Vienna, Waehringer Guertel 18–20, 1090 Vienna, Austria; 2 Department for Radiology, Medical University of Vienna, Vienna, Austria; 3 Center for Anatomy and Cell Biology, Medical University of Vienna, Vienna, Austria; 4 Center for Translational and Clinical Research, University of Zagreb, Zagreb, Croatia

**Keywords:** Bone ossification, Bone healing, CT scan analysis, Analysis of osteotomy gap, Radiographic analysis

## Abstract

**Background:**

The aim of this study was to analyze radiographic imaging techniques and to quantify bone ossification in the osteotomy gap after high tibial osteotomy.

**Material and methods:**

Study phase 1: high tibial osteotomy was performed on six lower extremities of human body donors and experimental X‑rays and computed tomography (CT) scans were applied. Different techniques were evaluated by three specialists for best representation of the osteotomy gap.

Study phase 2: optimized radiological techniques were used for follow-up on 12 patients. The radiographs were examined by 3 specialists measuring 10 different parameters. The CT scans were analyzed with semiautomatic computer software for quantification of bone ossification.

**Results:**

The osteotomy gap was best represented in 30° of flexion in the knee and 20° internal rotation of the leg. There were significant changes of the medial width over time (*p* < 0.019) as well as of the length of fused osteotomy, the Schröter score, sclerosis, trabecular structure and zone area measurements. Sclerosis, medial width of the osteotomy and area measurements were detected as reproducible parameters. Bone mineral density was calculated using CT scans, showing a significantly higher value 12 weeks postoperatively (112.5 mg/cm^3^) than at baseline (54.6 mg/cm^3^). The ossification of the gap was visualized by color coding.

**Conclusion:**

Sclerosis and medial width of the osteotomy gap as well as area measurements were determined as reproducible parameters for evaluation of bone healing. Quantification of bone ossification can be calculated with CT scans using a semiautomatic computer program and should be used for research in bone healing.

## Introduction

An open wedge osteotomy of the high tibia is a surgical technique to correct varus alignment of the lower limb caused by varus deformity of the tibia [1–3]. Osteosynthesis is performed using a locking plate to stabilize the osteotomy. The concept of osteotomy healing is well described in the literature [4]. If the gap is fixed with rigid immobilization, reconstruction of the cortex is achieved by radial filling of the gap with lamellar bone. Less rigid immobilization leads to fracture repair that is characterized by formation of callus and consists of fibrocartilage, fibrous tissue and hyaline cartilage. The limiting factor for full weight-bearing is ossification in the osteotomy gap since a failure of correction is associated with a collapse of the osteotomy gap due to malunion and plate failure [5–9]. Commonly used radiological imaging techniques fail to provide an exact representation of the osteotomy gap and thus the ossification of the gap. Analyzing bone healing in the osteotomy gap is not standardized either; however, there are attempts to develop a score regarding ossification of allografts which were inserted in the osteotomy gap [10–13].

The aim of this study was to analyze and quantify ossification of the osteotomy gap based on conventional radiography and computed tomography (CT) scans and to develop radiological imaging techniques for exact representation of the osteotomy gap. In this study, no allografts or autografts were used to fill the osteotomy gap.

## Material and methods

The study was conducted in two phases. Phase 1 used experimental radiological imaging, which was performed on the lower extremities of human body donors to evaluate the best radiological method for exact representation of the osteotomy gap. Phase 2 used the X‑ray technique, which provided the most promising results and then applied it in a study of 12 patients.

### Study phase 1

The study protocol was approved by the institutional ethics committee (Ethics Committee Number 1601/2012). A total of six lower extremities of human body donors were provided by the Center for Anatomy and Cell Biology: the mean age of the human body donors was 79 years and there were 4 male and 2 female donors. Inclusion criteria were intact knee joints, tibia, femur and soft tissue, no previous surgical treatment and no implants. After performing the osteotomy, X‑rays and CT scans were performed.

The plates and locking screws used for this study were Tomofix plates (Synthes, Johnson & Johnson, West Chester, Pennsylvania, USA). The Tomofix medial high tibia plate is designed according to the principles of the locking compression plate (LCP). For this study, the standard sized plate 440.834S Tomofix (pure titanium) was used. The plate measures 115 mm in length, 16 mm width and 3 mm thick and has 4 threaded holes proximal and 4 distal holes. The operation was performed as described in the technique guide of Synthes GmbH and as performed in daily clinical work. Of the six specimens one had to be excluded from evaluation due to a fractured lateral cortex.

#### Radiological investigation

The X‑rays were performed in specific positions with the help of a synthetic splint. To assure a strict side profile view of the plate, all human specimens were fixed in an internal rotation. Anteroposterior (AP) images were taken with the aid of synthetic wedges and a goniometer in 0°, 10°, 20°, 30° and 40° of flexion, respectively; lateral images were taken in the standard technique used in clinical routine. The CT scans (Philips Brilliance, Amsterdam, Netherlands) were then performed using a model 3CT calibration phantom (MindWays™, Austin, TX, USA) that was placed under the specimen.

**Fig. 1:**
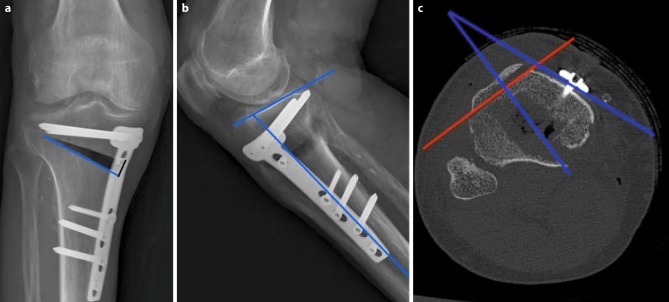
Measurement techniques on length of osteotomy (*blue line*) and medial osteotomy width (*black line*) are displayed in picture **a**; measurement technique of tibial slope and osteotomy slope (*blue lines*) are shown in picture **b**. In axial CT sequences, rotational offset of the plate (referring to the tibial tuberosity) was measured (*red lines*) (**c**)

Radiological parameters were measured by one specialist (orthopedic surgeon) as follows: in the CT scans, the position of the plate was determined in the axial images by measuring the rotational offset of the plate referring to the tibial tuberosity as 0° (see Fig. 1). The medial osteotomy width, tibial slope and osteotomy slope were measured on the X rays. Furthermore, the representation of the osteotomy was evaluated by judging the bony overlap of the osteotomy borders into the osteotomy gap (yes/no) at different degrees of flexion.

### Study phase 2

The 12 patients (10 male/2 female patients) who underwent high tibial osteotomy (HTO) were included in a prospective study to apply the developed techniques in humans (Ethics Committee Number 1047/2013). Inclusion criteria were age (18–50 years old), not pregnant and no previous operations of the operated leg. The mean age was 43.1 years, comorbidities were smoking (1 smoker) and pre-obesity (mean body mass index, BMI of 27.4) as well as obesity (1 patient with a BMI of 40). All patients provided written informed consent. The surgical technique was the same as in the cadaver model. The X‑rays were taken 1 day after the operation, 6 weeks and 12 weeks postoperatively. The CT scans were conducted 1 day after surgery and 12 weeks postoperatively. The Model 3CT Calibration Phantom (MindWays™) was used for all CT scans. The X‑rays were taken in the ideal position of the leg that had been determined from the cadaver model.

#### Evaluation of radiographs

**Fig. 2:**
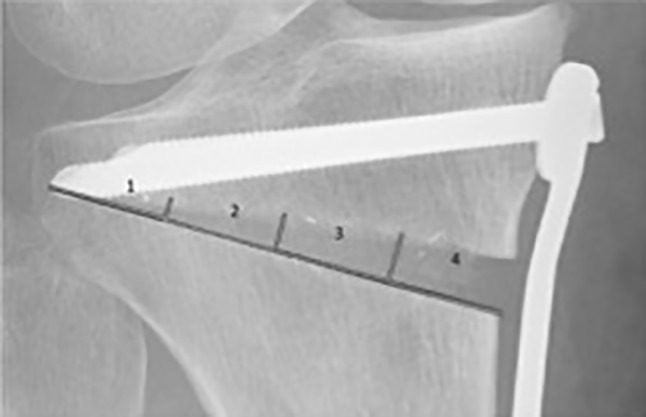
Division of gap into 4 zones for further radiological evaluation

All radiographs were analyzed by three specialists (orthopedic surgeons). The analysis included assessment of the osteotomy gap (osteotomy length, length of fused osteotomy, medial width of osteotomy), sclerosis (0 = no sclerosis, 1 = mild sclerosis, thinner than half of the lateral cortex, 2 = sclerosis, equal or more than half of the thickness of the lateral cortex), trabecular structure (0 = none, 1 = sparse growth, 2 = trabecular ossification) and the score for gap healing as described by Schröter et al. [14]. For this score, the osteotomy length (A) and the length of the fused osteotomy are measured (B). The percentage of gap filling with new bone is the result of dividing distance B by distance A. To evaluate ossification and morphology of bone healing, the osteotomy gap was divided into four zones numbered from lateral to medial osteotomy site (see Fig. 2). In each zone, the non-ossified area was measured, using the imaging software Impax ® EE (Agfa HealthCare GmbH, Bonn, Germany).

#### Semi-automatic evaluation of computed tomography (CT) images

**Fig. 3:**
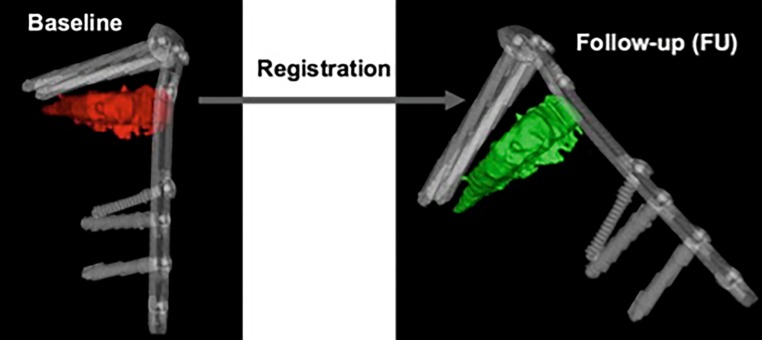
The osteotomy wedge is segmented manually at baseline (*red*) and registered automatically at follow-up (*green*) examinations using the plate and screws as reference points

The CT scans were analyzed by using an in-house computer software, programmed in MATLAB 2014b (MathWorks, Natick, MA, USA) for three-dimensional visualization that enables estimation of the volumetric bone mineral density (BMD). The analysis was done in three steps:

1. Manual segmentation of the osteotomy wedge: the osteotomy wedge was manually segmented at baseline (i.e. initial time point) using ITK Snap [15]. Since the automatic algorithm cannot distinguish between bone marrow and blood because they have similar intensities (measured in Hounsfield units, HU), an automatic algorithm would fail and it was necessary to proceed with a precise manual segmentation of the osteotomy gap.
2. Segmentation of the plate and correspondence between two time points: for registering the two different time points an automatic extraction of the splint is needed (i.e. the Tomofix plate). Due to the high intensities of the splint, a simple thresholding approach was used. The threshold was set at 0.9 using a normalized CT, which is an intensity image containing values in the range 0–1. The range of these values corresponds to the minimum and maximum HU values in the CT image. With this method only the extraction of the Tomofix plate including the screws as a splint mask was achieved. The extracted mask was used for registration. Each splint mask was registered at each follow-up time point to the initial time point using the Elastix toolbox [16]. A three-dimensional (3D) rigid set registration algorithm with six degrees of freedom was used. Additionally, a cubic, multiresolution (four resolutions, with a mean of squared differences similarity) measure was used. This way, the already segmented osteotomy wedge mask at baseline could be transferred according to the position of the plate and to the corresponding follow-up CT image series using the transformation matrix of the registration algorithm. For every follow-up examination obtained 12 weeks postoperatively, the exact location of the wedge from the initial time point was determined and mapped (see Fig. 3).
3. Quantification of bone growth: based on the segmentation mask of the manually segmented osteotomy wedge, the local change of the wedge is compared for each voxel and changes in BMD are displayed. A Model 3CT Calibration Phantom (Mindways, Austin, TX, USA) was used to estimate bone mineral density. Through the reference material embedded in the phantom and the calibration information, it is possible to measure similar densities in unknown objects and indirectly measure BMD. Every voxel (HU) is converted with the help of the CT phantom into dipotassium phosphate (K_2_HPO_4_) equivalent bone mineral density values (mg/cm^3^) [17]. Density estimates derived from using the phantom should not be interpreted as “physical” units, but as relative values and are thus not displayed as units in the further text.

### Statistics

For statistical analysis, the program SPSS Version 23.0.0.0 (IBM, Armonk, NY, USA) was used. To evaluate the significance of the changes in BMD, a paired t‑test was performed after using the Kolmogorov-Smirnov test to evaluate the statistically normal distribution. For evaluation of changes in radiological parameters over time, a one-way ANOVA as well as post hoc tests (Bonferroni) were used. For radiological parameters with inhomogeneous variances, the Friedman test as well as the Wilcoxon test were used. The Friedman test was performed to evaluate if there were significant interobserver differences in all radiographic measurements, *p*-values <0.05 were considered to indicate statistical significance. To evaluate a correlation between changes in BMD and medial osteotomy width, the Pearson correlation coefficient for bivariate correlation was calculated.

## Results

### Study phase 1

**Fig. 4:**
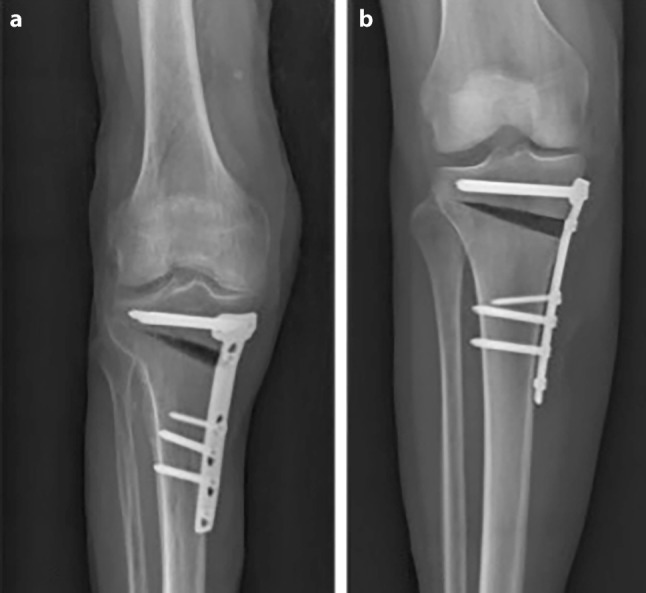
**a** The conventional AP radiography of the cadaveric leg shows tibial overlap of the posterior tibia in the gap (**b**) exact representation of the osteotomy gap is achieved with 20° of internal rotation and 30° of flexion in the knee joint

**Fig. 5:**
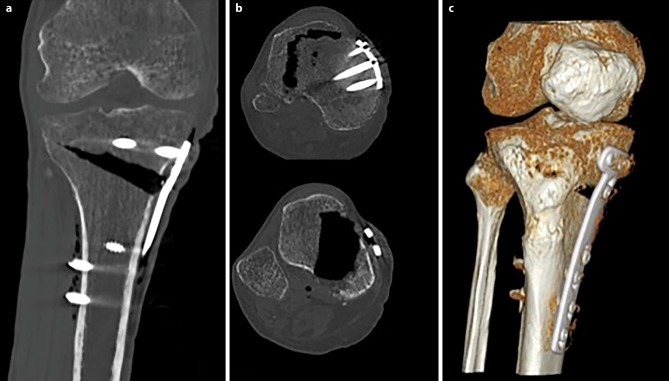
CT scans of cadaveric leg can bypass the obstacle of the overlap of the tibial tuberosity into the osteotomy gap (**a** shows an AP section image, **b** shows an axial section image and **c** shows a 3D reconstruction)

To establish exact representation of the osteotomy gap, a strict side profile view of the plate was achieved by internal rotation of the leg as well as flexion of the knee to compensate for the posterior slope of the tibia and the osteotomy itself (see Fig. 4). Best results were obtained with 30° of flexion (see Table 1). The mean osteotomy width was 12.5 mm (range 10.7–13.9 mm). The mean tibial slope was 10.3° (range 7.4–17.2°) and mean osteotomy slope was 17.2° (range 9.2–22.7°). The internal rotation offset of the plate measured in the axial CT scans showed a mean value of 25° (range 15–31.5°). The overlap of the tibial tuberosity is an obstacle for the evaluation of ossification in the central part of the gap, which can be overcome in the CT analysis (see Fig. 5). The CT scan was able to erase most of the metal artefacts and trabecular structure could be additionally displayed.

**Table 1:** Radiological measurement on human specimens showing osteotomy width, tibial and osteotomy slope as well as rotational offset of the plate and tibial overlap at different degrees of flexion

Specimen	Internal rotation of plate	Osteotomy width	Tibial slope	Osteotomy slope	Tibial overlap in flexion of the knee in				
	(°)	(mm)	(°)	(°)	0°	10°	20°	30°	40°
1	22.3	13.9	7.7	22.7	Yes	Yes	Yes	*No*	Yes
2	15	13.2	7.4	9.2	Yes	Yes	No	*Yes*	Yes
3	23.9	10.9	17.2	16	Yes	Yes	Yes	*No*	Yes
4	32.5	10.7	9.7	17.9	Yes	Yes	Yes	*No*	Yes
5	31.6	12	9.3	20	Yes	Yes	Yes	*No*	Yes
Mean	25.1	12.1	10.3	17.2	–	–	–	–	–

### Study phase 2

#### Radiography

**Fig. 6:**
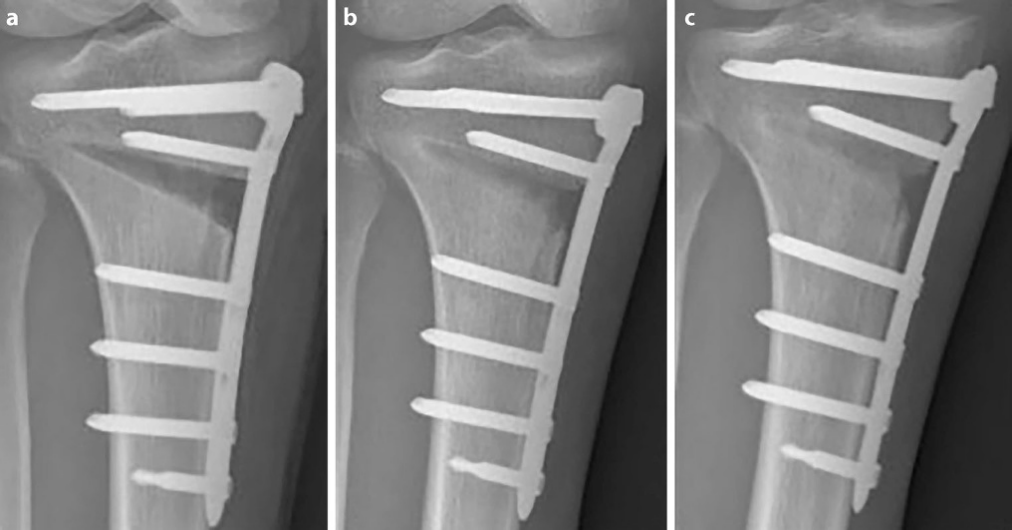
Radiological appearance of the osteotomy gap. **a** postoperatively, **b** 6 weeks postoperatively and **c** 12 weeks postoperatively with new bone healing spreading from lateral to the medial osteotomy side. The gap is visualized perfectly by flexion and inward rotation of the knee to rule out the overlap of the osteotomy gap and the plate

When applying the X‑ray technique optimized in the cadaver (30° of flexion and 25° of internal rotation of the knee joint), 21 out of 36 X‑rays showed exact visualization of the osteotomy gap (Fig. 6).

Mean osteotomy length was 73.34 mm (62.3–83.3 mm), mean osteotomy width was 10.84 mm (7.4–16.7 mm) (Table 2). When values were compared over time, there were statistically significant differences in medial width of osteotomy (*p* < 0.019), length of fused osteotomy (*p* < 0.001), the Schröter score (*p* < 0.001), sclerosis (*p* < 0.001) and trabecular structure (*p* < 0.001) as well as in area measurements (Table 2). Regarding the post hoc tests, significant changes in medial width of osteotomy and zone area measurements occurred between day 1 and week 12, but not between day 1 and week 6 or week 6 and week 12. Significant changes between all time points occurred in length of fused osteotomy, Schröter score and trabecular structure. There were no significant changes in length of osteotomy over time.

**Table 2:** Results of evaluation of the X‑rays and comparison of values between different time points. Values of parameters are expressed as means (and range); for homogeneous variances, a one-way ANOVA and post hoc tests were used. For parameters with inhomogeneous variances (sclerosis and trabecular structure), the Friedman test as well as the Wilcoxon test were used. Significant *p*-values (*p*-value <0.005) are marked in bold type

Parameter	Mean (range)			ANOVA/Friedman	Post hoc tests	(Bonferroni/Wilcoxon)	
				*p*-value	*p*-value		
	Day 1	Week 6	Week 12		Day 1: week 6	Week 6: week 12	Day 1: week 12
Length of osteotomy	73.34 mm	72.68 mm	72.61 mm	0.945	1.000	1.000	1.000
	(62.3–83.3 mm)	(61.4–83.8 mm)	(63–82.9 mm)	–	–	–	–
Length of fused	14.21 mm	37.82 mm	50.68 mm	**0.000**	**0.000**	**0.001**	**0.000**
Osteotomy	(3.3–29.2 mm)	(22.3–51.7 mm)	(24.8–66.1 mm)	–	–	–	–
Width of osteotomy	10.84 mm	9.58 mm	8.18 mm	**0.019**	0.467	0.397	**0.015**
	(7.4–16.7 mm)	(7.6–13.6 mm)	(6.1–10.9 mm)	–	–	–	–
Schröter score	19.14%	52%	70.6%	**0.000**	**0.000**	**0.008**	**0.000**
	(4.4–34.8%)	(36–72.1%)	(36.3–96.1%)	–	–	–	–
Zone 1	19.2 mm^2^	10.1 mm^2^	1.6 mm^2^	**0.006**	0.270	0.275	**0.004**
	(0–51.2 mm^2^)	(0–31.8 mm^2^)	(0–14.1 mm^2^)	–	–	–	–
Zone 2	64.9 mm^2^	49.2 mm^2^	20.2 mm^2^	**0.004**	0.663	0.082	**0.003**
	(6.9–123.4 mm^2^)	(9.4–106.8 mm^2^)	(0–77.1 mm^2^)	–	–	–	–
Zone 3	92.6 mm^2^	68.2 mm^2^	35.8 mm^2^	**0.003**	0.339	0.115	**0.002**
	(29.3–154.1 mm^2^)	(17.8–118.3 mm^2^)	(0–88.4 mm^2^)	–	–	–	–
Zone 4	150.4 mm^2^	115.3 mm^2^	80.6 mm^2^	**0.000**	0.082	0.086	**0.000**
	(68.3–284.6 mm^2^)	(51.7–156.2 mm^2^)	(34–148 mm^2^)	–	–	–	–
Sclerosis	–	–	–	**0.000**^**a**^	0.235^b^	**0.006**^**b**^	**0.072**^b^
	(0–1)	(0–1)	(0–2)	–	–	–	–
Trabecular	–	–	–	**0.000**^**a**^	**0.016**^**b**^	**0.005**^b^	**0.002**^**b**^
Structure	0	(0–1)	(1–2)	–	–	–	–

^a^Calculated using the Friedman test

^b^Calculated using the Wilcoxon signed ranks test

There were statistically significant differences between observers for length of osteotomy at day 1 (*p* = 0.038), length of fused osteotomy at day 1 and week 6 (*p* < 0.001 and *p* = 0.002), trabecular structure at week 6 (*p* = 0.025), the Schröter score at day 1 and week 6 (*p* < 0.001 and *p* = 0.002) as well as for zone 3 non-ossified area measurements at week 1 (*p* = 0.038) and zone 4 non-ossified area measurements at week 6 and week 12 (*p* = 0.027 and *p* = 0.027). There were no significant differences between other radiological measurements (Table 3). There was no correlation between changes in BMD over time and length of osteotomy (*p* = 0.983; Pearson correlation coefficient = 0.007).

**Table 3:** Interobserver differences of radiological measurements were calculated using the Friedman test. The median (50th percentile) is displayed for each observer. *P*-values <0.05 are marked in bold numbers

Parameter		Observer 1	Observer 2	Observer 3	*p*-value
Length of osteotomy	Day 1	70.250	77.850	72.650	**0.038**
	Week 6	70.300	75.850	71.350	0.191
	Week 12	69.00	74.000	72.250	0.108
Length of fused osteotomy	Day 1	7.150	16.050	16.300	**<0.001**
	Week 6	28.100	44.750	35.550	**0.002**
	Week 12	43.150	59.750	58.550	0.191
Medial width of osteotomy	Day 1	11.000	11.100	11.350	0.525
	Week 6	7.950	9.100	9.300	0.751
	Week 12	7.900	8.500	7.800	0.406
Non-ossified area					
Zone 1	Day 1	13.250	15.500	14.450	0.819
	Week 6	2.750	7.600	8.600	0.321
	Week 12	0.000	0.000	0.000	0.111
Zone 2	Day 1	70.850	65.350	63.500	0.382
	Week 6	56.000	38.200	35.800	0.275
	Week 12	8.700	10.000	9.950	0.547
Zone 3	Day 1	129.500	56.600	69.200	**0.038**
	Week 6	80.450	32.400	57.350	0.080
	Week 12	38.600	15.150	25.200	0.906
Zone 4	Day 1	151.000	161.400	151.650	0.080
	Week 6	116.500	129.350	98.550	**0.027**
	Week 12	73.550	87.500	67.850	**0.027**
Sclerosis	Day 1	0	0	0	0.368
	Week 6	0.50	0.00	0.50	0.815
	Week 12	1.00	1.00	1.00	0.671
Trabecular structure	Day 1	0.00	0.00	0.00	<0.900
	Week 6	0.00	0.00	1.00	**0.025**
	Week 12	1.00	1.00	1.00	0.510
Schröter score	Day 1	11.350	19.700	21.050	**<0.001**
	Week 6	36.650	61.000	55.200	**0.002**
	Week 12	63.150	79.400	78.000	0.249

#### CT scans

**Fig. 7:**
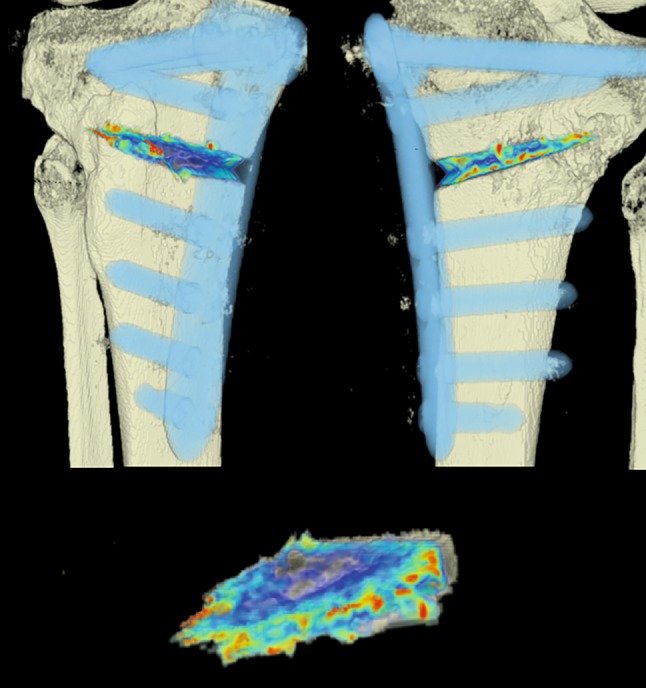
CT scan analysis included a visualization of the highest BMD changes: the gap is evenly filled with new bone; *red zones* mark the areas with most bone growth, *green zones* moderate bone growth and *blue areas* show only minimal changes in BMD

**Fig. 8:**
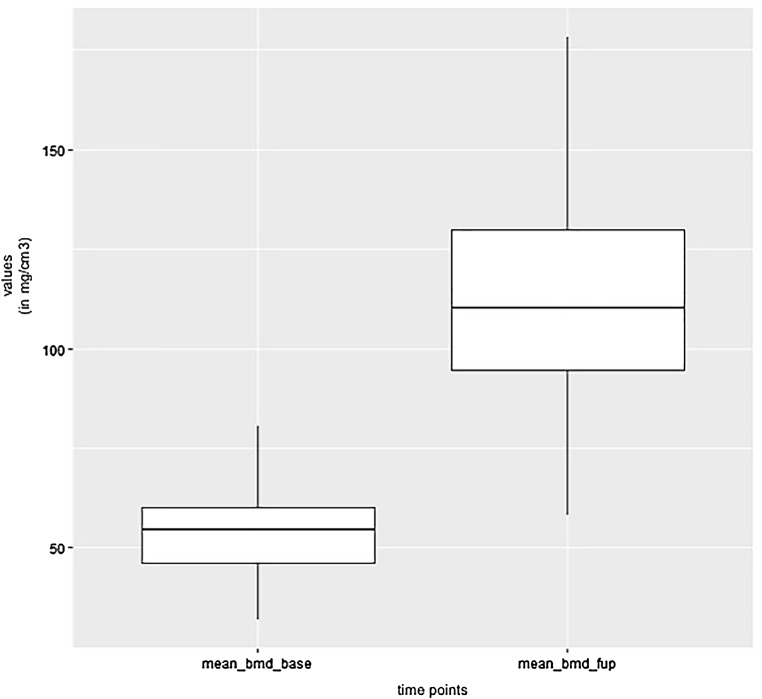
Box plot showing a significant increase of BMD after 12 weeks (*p* < 0.0002)

The areas with the greatest change of BMD representing the morphology of bone healing in the gap were visualized using color coding. Red zones mark the areas with most bone growth, green zones moderate bone growth and blue areas show only minimal changes in BMD (Fig. 7). Mean bone density in the osteotomy gap was significantly higher (*p* < 0.0002) 12 weeks postoperatively when compared to the baseline BMD calculated from the CT scans directly after the operation. The mean BMD at baseline was 54.586 mg/ccm (31.892–80.668) and increased to a mean BMD of 112.487 mg/ccm (58.141–178.214) at follow-up. The BMD increased in all patients except for one (Fig. 8 and Table 4).

**Table 4:** Mean BMD measurement values in Hounsfield units (HU) at baseline and follow-up; *p* < 0.0002. Visualization of changes in BMD for each patient are displayed using color coding (*red* = high bone growth, *green* = moderate bone growth, *blue* = low bone growth)

Patient	Mean BMD base	Mean BMD follow-up	Visualization of BMD changes
1	80.668	133.284	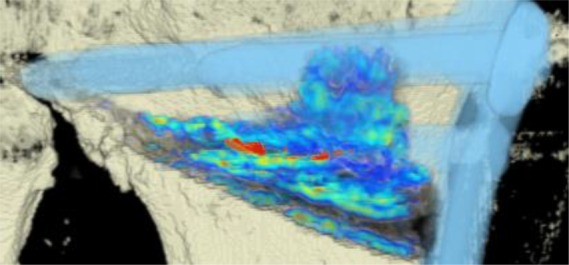
2	66.473	120.400	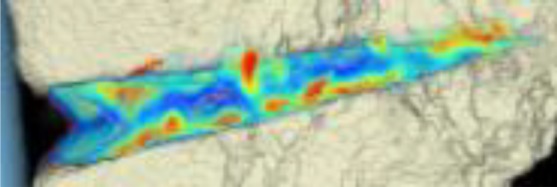
3	56.267	109.557	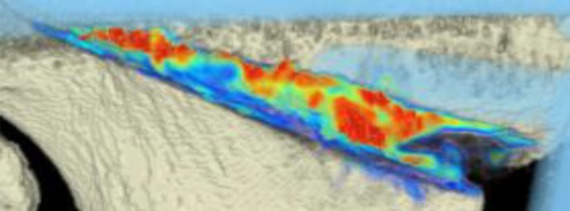
4	52.394	74.488	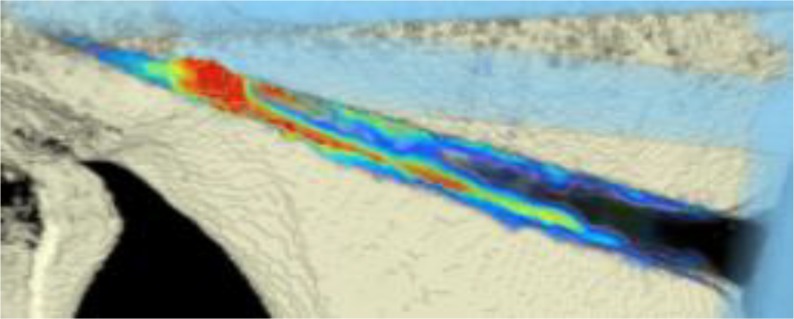
5	31.892	128.767	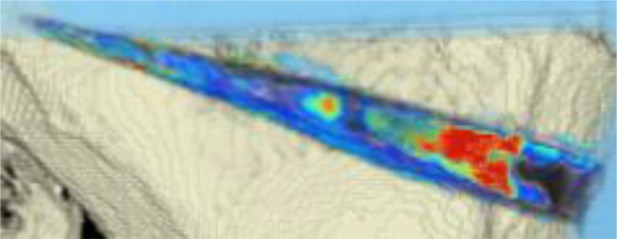
6	60.847	76.738	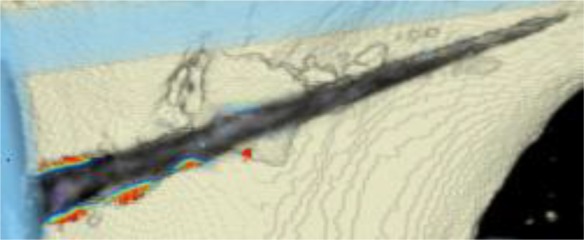
7	52.735	110.897	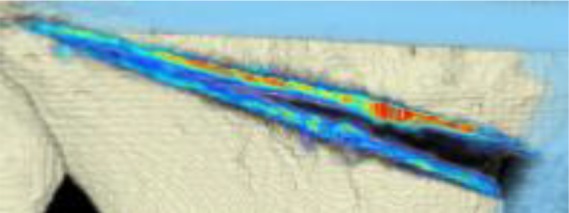
8	58.040	178.214	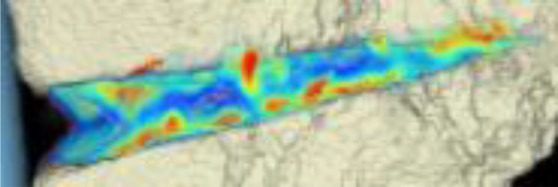
9	46.363	100.354	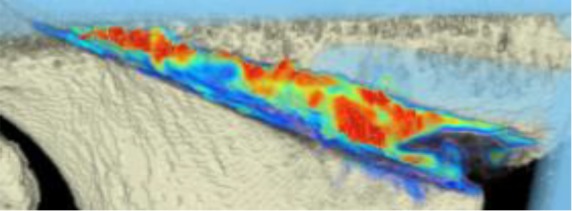
10	43.953	150.971	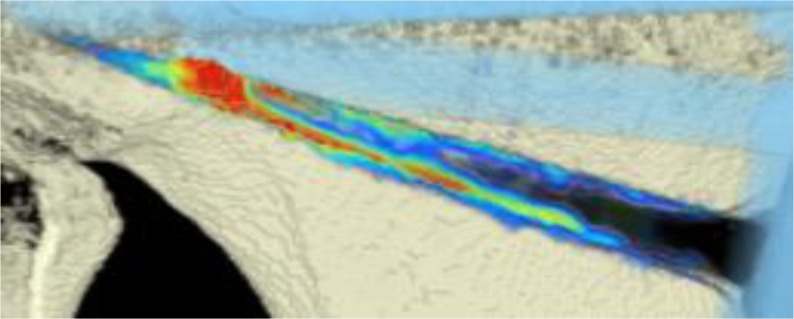
11	45.303	108.430	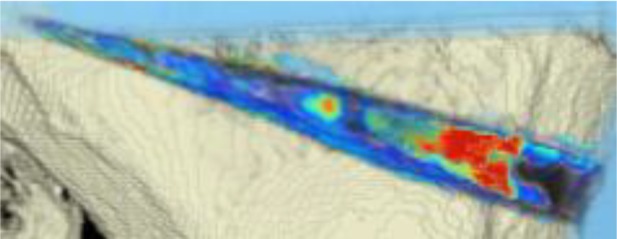
12	60.094	58.141	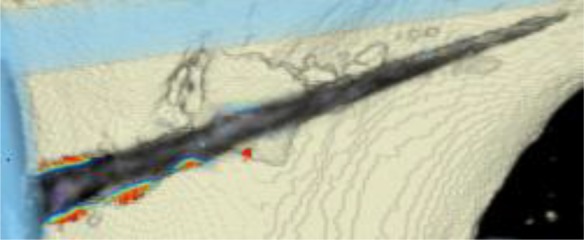
Mean BMD (range)	54.586 (31.892–80.668)	112.487 (178.214–58.141)	*p* < 0.0002

## Discussion

There have been attempts to quantify bone healing in osteotomy gaps. Van Hemert et al. graded bone healing and remodeling of allografts in the osteotomy gap in five phases [12]. These phases were adapted accordingly to the existing fracture healing phases first described by McKibbin [18]. Oh et al. described an adaption of the van Hemert score for bone and allograft healing in the gap, dividing the gap into four zones and starting the staging from the medial site [11]; since we did not use allografts in our study, this score was not applicable. There has been a study in which Akiyama et al. applied CT scans for follow-up in HTO patients with autologous osteophyte grafting but did not describe any evaluation system for gap healing [10]. Several recent studies examined HTO results with CT scans [19–21] using a descriptive evaluation of bone healing without quantification. There were several studies from Brinkman et al. [22], Schröter et al. [14] and Röderer et al. [23] describing a similar radiological assessment of bone filling in the osteotomy gap of HTO patients. We used the Schröter score for our study, as it appears to fulfil our aim to quantify bone healing in the gap. In their study, Schröter et al. did not present any grading of their results. Additionally, only one observer evaluated all radiographs.

The osteotomy gap could be represented well with our imaging technique by positioning the knee in an inward rotation and flexion; however, an exact representation was not possible in every patient. This is due to the fact that every HTO is performed depending on individual tibial slope and anatomical situation. The amount of internal rotation and flexion of the knee we described can therefore be used as a recommendation and should be adapted for each patient with the help of a C-arm.

Over time, there was a significant change in medial width of osteotomy as well as in all area measurements, which confirms that the osteotomy gap narrowed over time. These findings were objectively supported by the CT scan results, where changes in BMD are displayed all over the osteotomy surfaces. New bone on the edges of the osteotomy surfaces was not visualized clearly on plain radiographs. What appeared to be sclerosis on the osteotomy surfaces proved to be new bone in the CT. This hypothesis is also supported by the fact that sclerosis assessments significantly changed between measured time points. With these findings, sclerosis and medial width of the osteotomy could be used as reliable parameters for ossification in the judgment of conventional X‑rays. Those two parameters were the most reproducible out of the 10 parameters evaluated. We believe that the reason for the accuracy is that these measurements are easier to take; this is because the observers do not need to quantify bone healing directly, but indirectly. When it comes to the Schröter score and the length of fused osteotomy (which is used for calculating the score), there are significant interobserver deviations. When looking at *p*-values for both parameters (Schröter score and length of fused osteotomy), observers seemed to agree only at week 12. The Schröter score requires assessment of bone healing for measuring the length of fused osteotomy and thus failed as every observer assessed the fusion differently at day 1 and week 6. At week 12, observers agreed upon fusion because bone healing was more visible compared to the time points before. Interestingly, area measurements in zones 1 and 2 showed no significant differences between observers, this could be due to the fact that both zones do not include the tubercle of the tibia and are thus more easily visible than in zones 3 and 4. As zone 4 is the biggest zone to evaluate, differences were found here as well. In summary, we cannot recommend using area measurement and the Schröter score because of its incomplete and differing results.

With the help of the CT scan data, a computerized program was able to quantify the ossification of the gap. This is the first study to use an objective method for quantification of ossification in the osteotomy gap after HTO. Surprisingly, the morphology of bone healing was not as expected and described in literature [2, 3]. When comparing the CT scans to the radiographs, the radiographs suggested that bone healing mostly happens at the lateral osteotomy gap and from there grows to the medial side of the gap. When analyzing the CT scans and marking the areas with the greatest changes of bone density, we were able to see that new bone was growing on all osteotomy surfaces evenly throughout the gap. With our semi-automatic computer program, it was possible to quantify new bone formation by measuring bone mineral density and its changes over time. To our knowledge, there is no existing comparable quantification system for analysis of gap healing in the literature.

There are still shortcomings to this new quantification program. Since it is only semi-automatic, the first CT scans have to be manually segmented which is time consuming and requires some expertise. Accurate registration of the osteotomy surfaces at baseline is important to detect small changes and bone growth, which would be missed if a wrongly segmented wedge is transferred on the follow-up scan. We are working on the development of a fully automatic segmentation technique, which will eradicate the difficulties of manual segmentation.

## Conclusion

The use of CT scans and 3D analysis of the osteotomy gap adds important information to the understanding of the ossification process after HTO. Conventional radiography evaluation is associated with many interobserver differences. Assessment of sclerosis in the osteotomy gap as well as measurement of the medial width of the osteotomy, both qualified as reliable parameters for ossification. In this small patient cohort, the Schröter score for gap healing failed to provide exact estimation of bone growth when compared to CT results. We developed a new method for objective quantification of ossification in the osteotomy gap. The CT scans and the presented semi-automatic computer-assisted program should be used in further studies on bone formation in the osteotomy gap. The morphology of ossification in the gap was visualized well with the program. It showed that bone growth starts evenly on all osteotomy surfaces, thus narrowing the gap over time.

## References

[1] Iorio R, Pagnottelli M, Vadala A, Giannetti S, Di Sette P, Papandrea P (2013). Open-wedge high tibial osteotomy: comparison between manual and computer-assisted techniques. Knee Surg Sports Traumatol Arthrosc.

[2] Staubli AE, De Simoni C, Babst R, Lobenhoffer P (2003). TomoFix: a new LCP-concept for open wedge osteotomy of the medial proximal tibia—early results in 92 cases. Injury.

[3] Aryee S, Imhoff AB, Rose T, Tischer T (2008). Do we need synthetic osteotomy augmentation materials for opening-wedge high tibial osteotomy. Biomaterials.

[4] Cheal EJ, Mansmann KA, DiGioia AM, Hayes WC, Perren SM (1991). Role of interfragmentary strain in fracture healing: ovine model of a healing osteotomy. J Orthop Res.

[5] Miller BS, Dorsey WO, Bryant CR, Austin JC (2005). The effect of lateral cortex disruption and repair on the stability of the medial opening wedge high tibial osteotomy. Am J Sports Med.

[6] Schroter S, Gonser CE, Konstantinidis L, Helwig P, Albrecht D (2011). High complication rate after biplanar open wedge high tibial osteotomy stabilized with a new spacer plate (Position HTO plate) without bone substitute. Arthroscopy.

[7] Yacobucci GN, Cocking MR (2008). Union of medial opening-wedge high tibial osteotomy using a corticocancellous proximal tibial wedge allograft. Am J Sports Med.

[8] Warden SJ, Morris HG, Crossley KM, Brukner PD, Bennell KL (2005). Delayed- and non-union following opening wedge high tibial osteotomy: surgeons’ results from 182 completed cases. Knee Surg Sports Traumatol Arthrosc.

[9] van den Bekerom MP, Patt TW, Kleinhout MY, van der Vis HM, Albers GH (2008). Early complications after high tibial osteotomy: a comparison of two techniques. J Knee Surg.

[10] Akiyama T, Okazaki K, Mawatari T, Ikemura S, Nakamura S (2016). Autologous Osteophyte grafting for open-wedge high tibial osteotomy. Arthrosc Tech.

[11] Oh KJ, Ko YB, Jaiswal S, Whang IC (2016). Comparison of osteoconductivity and absorbability of beta-tricalcium phosphate and hydroxyapatite in clinical scenario of opening wedge high tibial osteotomy. J Mater Sci Mater Med.

[12] van Hemert WL, Willems K, Anderson PG, van Heerwaarden RJ, Wymenga AB (2004). Tricalcium phosphate granules or rigid wedge preforms in open wedge high tibial osteotomy: a radiological study with a new evaluation system. Knee.

[13] Jung KA, Lee SC, Ahn NK, Hwang SH, Nam CH (2010). Radiographic healing with hemispherical allogeneic femoral head bone grafting for opening-wedge high tibial osteotomy. Arthroscopy.

[14] Schroter S, Freude T, Kopp MM, Konstantinidis L, Dobele S, Stockle U (2015). Smoking and unstable hinge fractures cause delayed gap filling irrespective of early weight bearing after open wedge osteotomy. Arthroscopy.

[15] Catterall WA (1991). Functional subunit structure of voltage-gated calcium channels. Science.

[16] Klein S, Staring M, Murphy K, Viergever MA, Pluim JP (2010). elastix: a toolbox for intensity-based medical image registration. Ieee Trans Med Imaging.

[17] Cann CE (1988). Quantitative CT for determination of bone mineral density: a review. Radiology.

[18] McKibbin B (1978). The biology of fracture healing in long bones. J. Bone Joint Surg. Br..

[19] Mucha A, Dordevic M, Hirschmann A, Rasch H, Amsler F, Arnold MP (2015). Effect of high tibial osteotomy on joint loading in symptomatic patients with varus aligned knees: a study using SPECT/CT. Knee Surg Sports Traumatol Arthrosc.

[20] Kim TW, Kim BK, Kim DW, Sim JA, Lee BK, Lee YS (2016). The SPECT/CT evaluation of compartmental changes after open wedge high tibial osteotomy. Knee Surg Relat Res.

[21] Moghtadaei M, Otoukesh B, Bodduhi B, Ahmadi K, Yeganeh A (2016). Evaluation of patellar position before and after medial opening wedge high tibial osteotomy: radiographic and computed Tomography findings. Med Arch.

[22] Brinkman JM, Lobenhoffer P, Agneskirchner JD, Staubli AE, Wymenga AB, van Heerwaarden RJ (2008). Osteotomies around the knee: patient selection, stability of fixation and bone healing in high tibial osteotomies. J Bone Joint Surg Br.

[23] Roderer G, Gebhard F, Duerselen L, Ignatius A, Claes L (2014). Delayed bone healing following high tibial osteotomy related to increased implant stiffness in locked plating. Injury.

